# Supplementary material for: Acquisition of auditory discrimination mediated by different processes through two distinct circuits linked to the lateral striatum
Source: eLife. 2025 Jul 11;13:RP97326. doi: 10.7554/eLife.97326 (PMC12252547; doi:10.7554/eLife.97326)
Supplement: Figure 2—source data 2. [file elife-97326-fig2-data2.docx]

**Task-related brain activity during the acquisition of auditory discrimination**.

| Learning days | Brain regions | Laterality | T-value (peak) | Volume* (mm^3^) |
| --- | --- | --- | --- | --- |
| 2 | *Cerebral cortex* |  |  |  |
|  | Entorhinal cortex | R | 5.52 | 4.75 |
|  |  | L | 8.48 | 11.18 |
|  | Perirhinal cortex | L | 4.71 | 0.20 |
|  | Primary somatosensory cortex, jaw region | R | -3.93 | 0.84 |
|  |  | L | -4.89 | 0.29 |
|  | Primary somatosensory cortex, barrel field | R | -5.36 | 0.84 |
|  |  | L | -4.97 | 0.20 |
|  |  |  |  |  |
|  | *Midbrain* |  |  |  |
|  | Inferior colliculus | L | -4.88 | 0.47 |
|  |  |  |  |  |
|  | *Medulla* |  |  |  |
|  | Inferior olive | R | 6.89 | 1.03 |
|  |  |  |  |  |
|  | *Cerebellum* |  |  |  |
|  | Copula of the pyramis | L | 5.01 | 0.59 |
|  | Crus 2 of the ansiform lobule | L | 6.51 | 0.85 |
|  | Cerebellar lobule 2 | R | -4.83 | 0.57 |
|  | Cerebellar lobule 7/8 | R | -5.57 | 0.37 |
| 6 | *Cerebral cortex* |  |  |  |
|  | Primary motor cortex | L | 6.48 | 3.59 |
|  | Secondary motor cortex | R | 5.80 | 1.32 |
|  |  | L | 5.77 | 3.59 |
|  | Perirhinal cortex | L | 4.75 | 0.25 |
|  |  |  |  |  |
|  | *Basal ganglia* |  |  |  |
|  | Anterior dorsolateral striatum | R | 6.70 | 1.99 |
|  |  | L | 4.76 | 0.63 |
|  | Posterior ventrolateral striatum | R | 4.51 | 0.21 |
|  | Nucleus accumbens | R | 4.50 | 0.44 |
|  | Substantia nigra pars compacta/reticulata | L | 5.61 | 1.54 |
|  |  |  |  |  |
|  | *Hippocampus* |  |  |  |
|  | Hippocampus CA1 field | L | 6.26 | 1.02 |
|  |  |  |  |  |
|  | *Thalamus* |  |  |  |
|  | Ventral posterolateral thalamic nucleus | L | 5.14 | 0.83 |
|  | Medial geniculate body | L | 5.23 | 0.49 |
|  |  |  |  |  |
|  | *Medulla* |  |  |  |
|  | Ventral cochlear nucleus | R | -7.16 | 16.45 |
|  |  |  |  |  |
|  | *Cerebellum* |  |  |  |
|  | Crus 1 of the ansiform lobule | R | -4.79 | 16.45 |
|  |  | L | -5.86 | 0.64 |
|  | Paraflocculus | R | -7.38 | 16.45 |
|  |  | L | -5.49 | 4.87 |
| 10 | *Cerebral cortex* |  |  |  |
|  | Agranular insular cortex posterior part | R | 4.25 | 0.15 |
|  |  | L | 4.60 | 0.73 |
|  | Entorhinal cortex | R | 8.05 | 4.12 |
|  | Orbitofrontal cortex | R | -7.24 | 1.81 |
|  |  |  |  |  |
|  | *Basal ganglia* |  |  |  |
|  | Posterior ventrolateral striatum | R | 7.30 | 0.43 |
|  |  | L | 5.57 | 2.57 |
|  | Substantia nigra pars compacta/reticulata | R | 4.35 | 1.40 |
|  |  |  |  |  |
|  | *Amygdala* |  |  |  |
|  | Central amygdaloid nucleus | R | 6.91 | 0.66 |
|  |  |  |  |  |
|  | *Thalamus* |  |  |  |
|  | Medial geniculate body | R | 5.41 | 0.24 |
|  |  | L | 4.39 | 0.11 |
|  | Mediodorsal thalamic nucleus | L & R | -6.82 | 5.59 |
|  | Ventral anterior thalamic nucleus | L & R | -6.08 | 5.59 |
|  | Habenular nucleus | L | -5.02 | 1.00 |
|  |  |  |  |  |
|  | *Midbrain* |  |  |  |
|  | Dorsal cochlear nucleus | L & R | 8.47 | 13.12 |
|  |  |  |  |  |
|  | *Medulla* |  |  |  |
|  | Pontine reticular nucleus | L | -5.47 | 1.28 |
|  | Dorsal tegmental nucleus | L | -5.92 | 13.41 |
|  |  |  |  |  |
|  | *Cerebellum* |  |  |  |
|  | Crus 1 of the ansiform lobule | L | -4.94 | 0.28 |
|  | Cerebellar lobule 3/4/6 | L | -7.88 | 13.41 |
|  | Cerebellar lobule 10 | L | -5.33 | 13.41 |
|  | Paraflocculus | R | -9.41 | 6.65 |
|  |  | L | -6.20 | 9.41 |
| 24 | *Cerebral cortex* |  |  |  |
|  | Prelimbic cortex | L & R | -6.26 | 2.19 |
|  | Agranular insular cortex | R | 6.24 | 22.36 |
|  |  | L | 7.10 | 25.85 |
|  | Cingulate cortex/Secondary motor cortex | L & R | -7.40 | 117.86 |
|  | Primary somatosensory cortex, barrel field | R | -4.59 | 0.22 |
|  |  | L | -5.40 | 117.86 |
|  | Primary somatosensory cortex, trunk region | R | -4.22 | 0.24 |
|  | Retrosplenial cortex | L & R | -8.19 | 117.86 |
|  | Temporal association cortex | R | 4.10 | 2.58 |
|  |  | L | 6.44 | 25.85 |
|  | Entorhinal cortex | L | -5.89 | 0.44 |
|  | Claustrum | R | 5.74 | 0.96 |
|  |  |  |  |  |
|  | *Basal ganglia* |  |  |  |
|  | Dorsomedial striatum | L | -6.94 | 117.86 |
|  |  |  |  |  |
|  | *Hippocampus* |  |  |  |
|  | Hippocampus CA1 field | R | -4.46 | 0.24 |
|  |  | L | -8.59 | 117.86 |
|  |  |  |  |  |
|  | *Thalamus* |  |  |  |
|  | Mediodorsal thalamic nucleus | L & R | -8.98 | 117.86 |
|  | Laterodorsal thalamic nucleus | L & R | -7.54 | 117.86 |
|  |  |  |  |  |
|  | *Midbrain* |  |  |  |
|  | Dorsal cochlear nucleus | L & R | 7.73 | 37.60 |
|  |  |  |  |  |
|  | *Pons* |  |  |  |
|  | Pontine nuclei | L & R | -6.21 | 1.34 |
|  | Locus coeruleus | R | -4.97 | 0.31 |
|  |  |  |  |  |
|  | *Medulla* |  |  |  |
|  | Medial mammillary nucleus | L & R | 8.47 | 37.60 |
|  |  |  |  |  |
|  | *Cerebellum* |  |  |  |
|  | Crus 1 of the ansiform lobule | L | 5.78 | 3.90 |
|  | Crus 2 of the ansiform lobule | R | 6.06 | 1.94 |
|  |  | L | 5.41 | 3.90 |
|  | Cerebellar lobule 3/4/6 | R | -7.82 | 4.69 |
|  | Cerebellar lobule 10 | R | -4.91 | 0.26 |
|  | Paramedian lobule | R | 4.36 | 0.23 |
|  | Paraflocculus | R | -7.48 | 117.86 |
|  |  | L | -9.90 | 9.17 |
|  | Lateral cerebellar nucleus | R | -7.74 | 117.86 |
|  |  | L | -6.46 | 9.17 |
|  | Medial vestibular nucleus | L | -5.37 | 0.49 |

Rats (*n* = 14) were trained using the single lever press task, followed by the auditory discrimination task. ^18^F-FDG-PET scanning was conducted on Day 4 of the single lever press task, and then during a series of auditory discrimination task on Days 2, 6, 10, and 24. The regional brain activity related to behavioral tasks was calculated with a voxel-based statistical parametric analysis that compared ^18^F-FDG images on the single lever press task with those on either day (Days 2, 6, 10, and 24) of the discrimination task. Task-related brain regions with greater (T-value > 0) or smaller (T-value < 0) activity are summarized. The brain activity was increased or decreased in various brain regions during the progress of auditory discrimination. Especially, the increased activity was observed in the medial geniculate body, which sends ascending information to the auditory cortex and also receives feedback corticothalamic projections (***Lee, 2013***). However, there was no significant changes in the brain activity in the auditory cortex, being consistent with the results of a previous work reporting that the auditory cortex does not show the correlation to the pure-tone discrimination task (***Gimenez et al., 2015***). R and L indicate right and left hemispheres, respectively. The value of 3.8 was used as the threshold corresponding to the *p* < 0.001 (uncorrected) threshold. *Original volume.

References

Gimenez TL, Lorenc M, Jaramillo S. 2015. Adaptive categorization of sound frequency does not require the auditory cortex in rats. *Journal of Neurophysiology* **114**:1137-1145. DOI: 10.1152/jn.00124.2015, PMID: 26156379

Lee CC. 2013. Thalamic and cortical pathways supporting auditory processing. *Brain and Language***126**:22-28. DOI: 10.1016/j.bandl.2012.05.004, PMID: 22728130
